# Evidence for densovirus integrations into tapeworm genomes

**DOI:** 10.1186/s13071-019-3820-1

**Published:** 2019-11-27

**Authors:** Michaela Herz, Klaus Brehm

**Affiliations:** 1 Institut für Hygiene und Mikrobiologie, Universität Würzburg, Josef-Schneider-Str 2/E1, 97080 Würzburg, Germany; 2 Konsiliarlabor für Echinokokkose des Robert-Koch-Instituts, Josef-Schneider-Straße 2/E1, 97080 Würzburg, Germany

**Keywords:** Echinococcus, Echinococcosis, Densovirus, Parvovirus, Mobile genetic element, Gene silencing, Stem cell, Epigenetic

## Abstract

**Background:**

Tapeworms lack a canonical piRNA-pathway, raising the question of how they can silence existing mobile genetic elements (MGE). Investigation towards the underlying mechanisms requires information on tapeworm transposons which is, however, presently scarce.

**Methods:**

The presence of densovirus-related sequences in tapeworm genomes was studied by bioinformatic approaches. Available RNA-Seq datasets were mapped against the *Echinococcus multilocularis* genome to calculate expression levels of densovirus-related genes. Transcription of densovirus loci was further analyzed by sequencing and RT-qPCR.

**Results:**

We herein provide evidence for the presence of densovirus-related elements in a variety of tapeworm genomes. In the high-quality genome of *E. multilocularis* we identified more than 20 individual densovirus integration loci which contain the information for non-structural and structural virus proteins. The majority of densovirus loci are present as head-to-tail concatemers in isolated repeat containing regions of the genome. In some cases, unique densovirus loci have integrated close to histone gene clusters. We show that some of the densovirus loci of *E. multilocularis* are actively transcribed, whereas the majority are transcriptionally silent. RT-qPCR data further indicate that densovirus expression mainly occurs in the *E. multilocularis* stem cell population, which probably forms the germline of this organism. Sequences similar to the non-structural densovirus genes present in *E. multilocularis* were also identified in the genomes of *E. canadensis*, *E. granulosus*, *Hydatigera taeniaeformis*, *Hymenolepis diminuta*, *Hymenolepis microstoma*, *Hymenolepis nana*, *Taenia asiatica*, *Taenia multiceps*, *Taenia saginata* and *Taenia solium*.

**Conclusions:**

Our data indicate that densovirus integration has occurred in many tapeworm species. This is the first report on widespread integration of DNA viruses into cestode genomes. Since only few densovirus integration sites were transcriptionally active in *E. multilocularis*, our data are relevant for future studies into gene silencing mechanisms in tapeworms. Furthermore, they indicate that densovirus-based vectors might be suitable tools for genetic manipulation of cestodes.
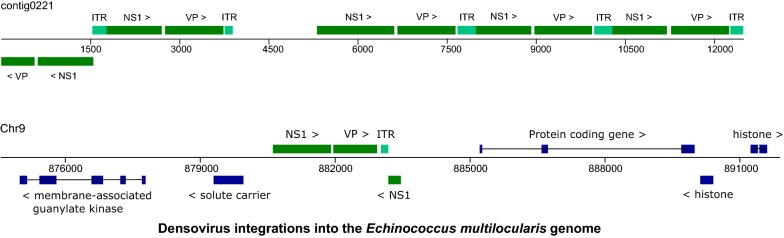

## Background

Tapeworms (cestodes) form a group of highly specialized, obligate endoparasites that display extreme features of adaptation to their hosts such as the complete loss of a gut and a highly modified, segmented, body plan [1]. The strobilar adult stages of cestodes typically reside in the intestine of vertebrates and their complex life-cycles comprise several ontogenetically distinct larval stages. Among the estimated 6000 tapeworm species, the three species, *Echinococcus multilocularis* (fox tapeworm), *E. granulosus* (dog tapeworm), and *Taenia solium* (pork tapeworm), are of particular medical and veterinary interest since their larval stages reside within the inner organs of humans and livestock animals, thus causing the diseases alveolar echinococcosis, cystic echinococcosis, and cysticercosis/neurocysticercosis, respectively [2, 3]. The combined global burden of these parasites is estimated to about 4.5 million DALYs (disability-adjusted-life-years-lost) annually in humans [4–6], with about 100,000 USD lifetime treatment costs for patients in developed countries, and about two billion USD annually for animal health costs [5]. In general, larval cestode infections are difficult to treat and, apart from surgical intervention, only very few antiparasitics (such as benzimidazoles) are currently available for chemotherapy [3, 7].

Towards a closer understanding of cestode biology, we and others have previously characterized the genomes of several cestode species, with the genome of *E. multilocularis* serving as a high-resolution reference [8, 9]. As a model system for adult cestodes, the dwarf tapeworm *Hymenolepis diminuta* and a number of additional model cestodes such as *Schistocephalus solidus* and *Mesocestoides corti* are currently being genomically and transcriptomically characterized [10]. A striking feature of cestode (and trematode) genomes is the absence of true *piwi* and *vasa* orthologues [8, 11], indicating that these organisms lack a canonical piRNA pathway which, in many other metazoans, mediates silencing of mobile genetic elements (MGE) [12] and is considered part of the hypothesized germline multipotency programm [13]. This raises questions concerning alternative MGE silencing pathways in cestodes [11], which, to be properly addressed, first require the characterization of repetitive elements in their genomes. With the exception of a few reports on repetitive elements encoding spliced leader RNAs [14, 15], inactive copies of Gypsy class Long Terminal repeats [16], and stem cell-specifically expressed copies of a TRIM (terminal repeat retrotransposon in miniature)-element [17]; however, respective information is presently scarce.

The virus family *Parvoviridae* contains the two subfamilies, *Parvovirinae* and *Densovirinae*, which infect vertebrates and invertebrates, respectively [18]. All parvoviruses have small, linear, single-stranded DNA genomes of about 5 kb, which encode two functionally different sets of polypeptides: the non-structural (NS) proteins which are necessary for viral gene expression and replication, and the structural proteins of the capsid (VP), which are often encoded by overlapping transcription units [19]. Best studied are the parvoviral NS1 proteins which belong to the superfamily 3 helicases and contain a conserved helicase domain that is essential for viral genome replication. Densovirus replication usually occurs within mitotically active host cells and originates from inverted terminal repeats of the virus DNA involving a rolling circle replication mechanism [20]. The transcription of virus genes within host cells is mostly directed by conserved promoter structures upstream of the viral coding sequences, and transcript processing as well as translation of viral proteins involves a number of different mechanisms such as alternative splicing, leaky scanning, and alternative initiation codon usage [21, 22]. Although the molecular mechanism of parvoviral integration into host DNA has not yet been studied in much detail, it is well established that parvovirus and densovirus sequences are widely distributed in vertebrate and invertebrate genomes and that these viruses cause a variety of pathologies from severe diseases to subclinical infections [23]. Interestingly, parvo- and densovirus vectors are currently also being developed as autonomously acting vehicles for genetic manipulation of several vertebrate and invertebrate species [24–26].

Apart from two anecdotal reports on the presence of parvovirus-like sequences in planarian and trematode genome assemblies [27, 28], no detailed analysis on possible parvo/densovirus sequence integration into flatworm genomes has yet been carried out. Based on the finding of densovirus-like sequences in the transcriptome of *E. multilocularis*, we herein carried out analyses on the presence of respective genes in the genome of this and other tapeworms. We provide evidence for densovirus sequences within the genomes of *Dibothriocephalus latus*, *Echinococcus canadensis*, *E. granulosus*, *E. multilocularis, Hydatigera taeniaeformis*, *Hymenolepis diminuta*, *H. microstoma*, *H. nana*, *Mesocestoides corti*, *Schistocephalus solidus*, *Spirometra erinaceieuropaei*, *Taenia asiatica*, *T. multiceps*, *T. saginata*, *T. solium* and *Schistosoma mansoni*. We also show that some of the integrated virus sequences are transcriptionally active in the *E. multilocularis* germinative cell population, which are mitotically active, pluripotent somatic stem cells that most probably form the germline of this organism. The majority of densovirus integration loci, however, is transcriptionally silenced. Our results are discussed in the background of future studies concerning gene silencing mechanisms in cestodes and the possible utilization of densovirus vectors for the development of transgenic methodology in these organisms.

## Methods

### Bioinformatic analysis

When mining the database WormBaseParaSite WBPS 10 [29–31] for viral genes in the *E. multilocularis* genome, we found the gene EmuJ_000388600, annotated as ‘non-capsid protein NS1’, which we analyzed further. Protein sequences for EmuJ_000388600 and the downstream open reading frame EmuJ_000388500 (downloaded from WormBaseParaSite WBPS 10 [29–31]) were used for BLASTP (E-value < 1e−10, identities > 20 %, coverage > 50%) searches against the SWISSPROT database at GenomeNET and domain analysis with pfam (E-value < 1e−10) [32]. A multiple sequence alignment was generated with the protein sequence for EmuJ_000388600 and its first two BLAST hits using MUSCLE v3.8.31 (4 iterations) [33, 34]. To detect further putative non-capsid protein 1 sequences in the *E. multilocularis* genome, we first performed BLASTP (E-value < 1e−10, identities > 80 %, coverage > 30%) searches against the protein predictions of *E. multilocularis* (downloaded from WormBaseParaSite WBPS 14 [29–31]) using EmuJ_000388600 as query. Sequences were retrieved and served as queries for BLASTP searches against the non-redundant sequences (nr) database at NCBI (E-value < 1e−10, identities > 90%, coverage > 90%). The confirmed sequences were utilized as query for TBLASTN (E-value < 1e−10, identities > 70%, coverage > 20%) searches against the *E. multilocularis* genome (downloaded from WormBaseParaSite WBPS 14 [29–31]). Non-redundant sequences were retrieved and confirmed by BLASTX searches against the non-redundant sequences (nr) database at NCBI (E-value < 1e−10, identities > 80%, coverage > 80%). For detailed analysis of densovirus integrations into the *E. multilocularis* genome, the sequences for the designated *Emu*DNV-NS1 (*E. multilocularis* densovirus non-capsid protein 1 gene) were curated individually, determining start and stop positions for the gene copies as well as their completeness. Frame shift mutations were identified by analysis of open reading frames (ORFs) using BioEdit six-frame translation [35]. In many cases, a second ORF downstream of *Emu*DNV-NS1 was detected. This ORF was presumed to be coding for a capsid protein (VP) and therefore designated *Emu*DNV-VP. The longest ORFs were used as query for BLASTN (E-value < 1e−10, identities > 90%, coverage > 10%) searches against the *E. multilocularis* genome to find additional gene copies. Detected *Emu*DNV-VP gene copies were curated individually as described for *Emu*DNV-NS1 and frameshift mutations were analyzed.

Protein structure analyses were performed with pfam (E-value < 1e−10) [32] using translated protein sequences of *Emu*DNV-NS1 and *Emu*DNV-VP. Protein sequences were also used for BLASTP (E-value < 1e−10, identities > 20%, coverage > 90%) searches against the SwissProt/UniProt database and non-redundant protein sequences (nr) database (organism viruses) at NCBI.

Inverted terminal repeats (ITRs) were identified with the computer program “einverted” (maximum extent of repeats 2000 bp, > 80% matches, loop < 100 bp) [36] using *Emu*DNV-NS1 nucleotide sequences together with 5000 bp flanking regions on both sides as input. To also discover remnants of ITRs nearby densovirus genes, local BLASTN (E-value < 1e−5, identities > 80%, coverage > 10%) searches against the *E. multilocularis* genome were performed with the longest identified ITR sequence. Densovirus loci were assessed by their genomic location using the genome browser Ensemble at WormBaseParaSite (WBPS10) [29–31]. Previous reports have identified putative TATA-boxes and activator elements for the *Penaeus stylirostris* densovirus [37]. We detected similar promotor structures for *Emu*DNV-NS1 and *Emu*DNV-VP by individual inspection of their upstream regions. Alignment of promotor regions was performed with MUSCLE (4 iterations) [33, 34].

For transcriptome data analysis, available RNA-Seq reads [8] (ENA sample accessions: ERS094035, ERS094036, ERS094037, ERS094038, ERS094039, ERS016464, ERS018054, ERS018053) were mapped to the *E. multilocularis* genome (downloaded from WormBaseParaSite WBPS7 [8, 29–31]) with Hisat2 v2.0.5 [38]. To discard all reads mapped to multiple genomic locations (mapping quality scores 0 and 1), only reads with a minimum quality score of 30 were counted using HTSeqCount v0.7.1 [39]. Expression levels were calculated as TPMs (Transcripts Per kilobase of exon per Million transcripts mapped).

To identify putative densovirus non capsid protein 1 gene sequences in other cestode genomes, we searched the genomes of *Dibothriocephalus latus* (D_latum_Geneva_0011_upd) [10], *Echinococcus canadensis* (ECANG7) [40], *E. granulosus* (EGRAN001 and ASM52419v1) [8, 9], *E. multilocularis* (EMULTI002) [8], *Hydatigera taeniaeformis* (H_taeniaeformis_Canary_Islands_0011_upd) [10], *Hymenolepis diminuta* (H_diminuta_Denmark_0011_upd) [10], *Hymenolepis microstoma* (HMN_v3) [8], *Hymenolepis nana* (H_nana_Japan_0011_upd) [10], *Mesocestoides corti* (M_corti_Specht_Voge_0011_upd) [10], *Schistocephalus solidus* (S_solidus_NST_G2_0011_upd) [10], *Spirometra erinaceieuropaei* (S_erinaceieuropaei) [41], *Taenia asiatica* (Taenia_asiatica_TASYD01_v1 and T_asiatica_South_Korea_0011_upd) [10, 42], *Taenia multiceps* (ASM192302v3) [43], *Taenia saginata* (ASM169307v2) [42], *Taenia solium* (Tsolium_Mexico_v1) [8], and as a trematode example, *S. mansoni* (Smansoni_v7) [44, 45] (downloaded from WormBaseParaSite WBPS 14 [29–31]) by local BLAST searches (for details on genomes see Additional file 1: Table S1). The putative non-capsid protein 1 EmuJ_000388600 served as query for TBLASTN searches against the downloaded genomes (E-value < 1e−5, identities > 30%, coverage > 30%). Non-redundant sequences were retrieved and utilized for reciprocal BLASTX searches against the non-redundant sequences (nr) database at NCBI (E-value < 1e−5, identities > 35%, coverage > 90%). Then, local BLASTN searches (E-value < 1e−10, identities > 70%, coverage > 30%) against the above-mentioned genomes were performed with the confirmed nucleotide sequences. To avoid retrieval of multiple sequences for the same gene copy, BLAST results overlapping more than 30% of their length were merged before sequence retrieval. Obtained sequences were verified by BLASTX searches against the non-redundant sequences (nr) database at NCBI (E-value < 1e−5, identities > 35%, coverage > 90%). Confirmed sequences with a coverage > 50% of the full-length gene version EmuJ_000388600 were used for phylogenetic analysis. Nucleotide sequences were aligned using MUSCLE in MEGA-X (align codons, 16 iterations) [33, 34, 46]. A bootstrap consensus tree was generated in MEGA-X [46] with the Neighbor-Joining method [47] using 1000 bootstrap replications [48] and pairwise deletion for gaps. Branches reproduced in less than 50% bootstrap replicates were collapsed. An overview of the bioinformatic workflow is shown in Additional file 2: Figure S1.

### Parasite material

Parasite material was maintained in Mongolian jirds (*Meriones unguiculatus*) by serial peritoneal passage as previously described [49, 50]. After isolation, parasite material was co-cultivated with rat Reuber hepatoma feeder cells [49]. For use in experiments, feeder cell-free metacestode or primary cell cultures were set up [49, 50].

### Hydroxyurea treatment of metacestodes

*In vitro* cultivated metacestode vesicles were treated with 40 mM hydroxyurea (HU) for 7 days as described previously [51]. Subsequently, metacestode vesicles were washed with PBS before RNA isolation. To monitor the success of the HU treatment, 2–3 vesicles of each cell culture flask were transferred to HU-free culture for 5-ethynyl-2′-deoxyuridine (EdU, Life Technologies, Darmstadt, Germany) incorporation, which was essentially performed as described previously using the short term labeling with 50 µM EdU for 5 hours [51]. Fluorescent detection of EdU was carried out with the Click-iT® EdU Alexa Fluor® 555 Imaging Kit (Life Technologies, Darmstadt, Germany) as described previously [51]. Samples were analyzed by epifluorescence microscopy (ZeissAxio Imager.Z1, Zeiss, Hamburg, Germany). The experiment was performed with three biological replicates.

### Primary cell culture

Feeder cell-free primary cell cultures were set up and cultivated for 2 days essentially as described previously [49, 50]. Primary cells were washed with PBS before RNA isolation.

### RNA isolation

Metacestode vesicles from HU treatment [51] were opened with a tip to disrupt the laminated layer and to remove cyst fluid. Primary cells and metacestodes were centrifuged at 500×*g* for 1 min. PBS was removed and the material was resuspended in 500 µl (cells) or 1 ml (vesicles) Trizol® Reagent (Invitrogen, Darmstadt, Germany), vortexed briefly and incubated at room temperature for 5 min. RNA extraction was performed using Direct-zol™ RNA MiniPrep (Zymo Research, Freiburg, Germany) according the manufacturer’s instructions (including DNase treatment).

### DNA isolation

Vesicles from feeder cell-free metacestode cultures were disrupted by pipetting, washed with PBS and centrifuged for 10 min at 5000×*g*. The supernatant was removed, and the pellet was re-suspended in lysis buffer (100 mM NaCl, 10 mM Tris-HCL (pH 8.0), 50 mM EDTA (pH 8.0), 0.5% SDS, 20 μg/ml RNase A, 0.1 mg/ml Proteinase K, 1.2 ml/100 mg pellet). After overnight incubation at 50 °C, a standard phenol-chloroform extraction was carried out, followed by an ethanol precipitation.

### Reverse transcription

Reverse transcription was performed with Omniscript® RT Kit (Qiagen, Hilden, Germany) or SuperScript®III Reverse Transcriptase (Invitrogen, Darmstadt, Germany) according to the manufacturersʼ instructions using an Oligo-dT primer (5′-ATC TCT TGA AAG GAT CCT GCA GGA CTT_22_VX-3′) or a combination of the Oligo-dT primer and a random octamer primer. An RT-neg control (no reverse transcriptase) was included for all samples.

### Cloning and sequencing

For the amplification of *Emu*DNV-NS1, primers were designed based on the sequences of the gene versions EmuJ_000034800, EmuJ_000388600, EmuJ_002195700 and EmuJ_000329200. PCR was performed on cDNA of 2-day-old primary cells using Taq-Polymerase (New England Biolabs, Schwalbach, Germany) with the primers 5′-GGC GTT CCA CTA CAA G-3′ and 5′-GCC AAC AAT TCA TAA ATG G-3′. RT-neg and gDNA controls were included. PCR products from cDNA were cloned into pJet1.2 using CloneJET^TM^ PCR Cloning Kit (Fermentas, St. Leon-Rot, Germany) and sequenced. The sequence of *Emu*DNV-NS1 was deposited at the EMBL Nucleotide Sequence Database under the accession number LR029140. To confirm the genome assembly at densovirus integration sites we performed PCR analysis and sequencing choosing primers annealing to an *Emu*DNV-NS1 gene version and to a neighboring tapeworm gene with annotated function. PCR was performed on gDNA using Taq-Polymerase (New England Biolabs, Schwalbach, Germany) with the primers 5′-GAT AGT CTG CCA TTA GGC-3′ and 5′-GGA AAC CTC CTC CGA CA-3′ for EmuJ_000013900; 5′-GCT TAT TCA TTC TGC GGT TTT-3′ and 5′-GAT AGT TTG TTC CAC CAT TGA-3′ for EmuJ_002195700; 5′-GAT TTC ATT GGC TGA AAA CAT-3′ and 5′-GGT GCT TTT TCA TAT TCT CGT-3′ for EmuJ_000388600; and 5′-GGC TCG AGG AAG GTA GTT GTC-3′ and 5′-GGC TCA ACA ACC GAC GTA AT-3′ for EmuJ_000329200. PCR products were cloned into pDrive Cloning Vector using the QIAGEN^®^ PCR Cloning Kit and sequenced.

### Quantitative real-time PCR

Quantitative real-time PCR was performed with StepOnePlus Real-Time PCR-Systems (Thermo Fisher Scientific, Schwerte, Germany). Primers for *Emu*DNV-NS1 were based on the sequences of the gene versions EmuJ_000034800, EmuJ_000388600 and EmuJ_000329200: 5′-CAA CCA GCA GGA TCT CAA GCA-3′ and 5′-CAT CTA CCC TCT ATG GCG GCT-3′. As the primers did not span an intron, RT-neg controls were used. *emelp* served as reference gene (primers: 5′-TGA TGA AAG TGA AGC CAA GGA ACT TGA G-3′ and 5′-TTC GTC TGG AGC GTC TCA TTC TTA GAG-5′). The following reaction mixture was used: 2 µl of 1:5 diluted cDNA (or RT-neg), 200 nM each primer (300 nM for *emelp*) and the HOT FIREPol®EvaGreen® qPCR Mix (ROX) (Solis Biodyne, Düsseldorf, Germany); with the following program: 15 min at 95 °C, 40 cycles of: 15 s at 95 °C, 20 s at 60 °C, 20 s at 72 °C; fluorescence measurement at 72 °C. Amplification product specificity was assessed by melting curve analysis and sequencing of the PCR-products. Experiment was performed with three technical and three biological replicates. The efficiency of the amplification was computed with linREG [52, 53]. For statistical analysis, relative gene expression was calculated using the formula of Pfaffl [54]. The permutation test was performed in fgStatisitics [55] with technical replicates sampled at random and 5000 resampling cycles.

## Results

### Identification of densovirus-derived genes in *E. multilocularis*

We previously established that growth and proliferation of the *E. multilocularis* metacestode stage is crucially driven by a population of pluripotent stem cells, called germinative cells, which are the only mitotically active cells in the metacestode [51] We also showed that around 25% of all cells of the metacestode are germinative cells and that this cell type is strongly enriched (up to 80%) in primary cell preparations of *E. multilocularis* [51]. In order to identify germinative cell-specifically expressed genes in the *E. multilocularis* genome we mined the database WormBaseParaSite WBPS 10 [29–31] and published transcriptome data [8] for genes enriched in primary cell preparations and identified one respective gene (EmuJ_000388600) which was annotated as ‘non-capsid protein NS1’. EmuJ_000388600 coded for a protein of 392 amino acids and, in BLASTP analyses against the SWISSPROT database, displayed highest similarities (~ 24% identical and 43% similar residues) to insect densovirus NS1 initiator proteins (see Additional file 3: Figure S2). Furthermore, when analyzed for conserved protein domains, a PPV_E1_C domain (Papillomavirus helicase E1 C-terminus) was identified the EmuJ_000388600 encoded protein. We thus concluded that EmuJ_000388600 probably resulted from an integration of a densovirus into the *E. multilocularis* genome and designated the gene *Emu*DNV-NS1. Immediately downstream of *Emu*DNV-NS1 we identified another reading frame (EmuJ_000388500) encoding a protein with weak homologies (below cut-off values) to the minor component of the viral capsid of the Pea enation mosaic virus, which further supported that we had identified a densovirus integration locus.

### Multiple densovirus integrations in the *E. multilocularis* genome

**Fig. 1:**
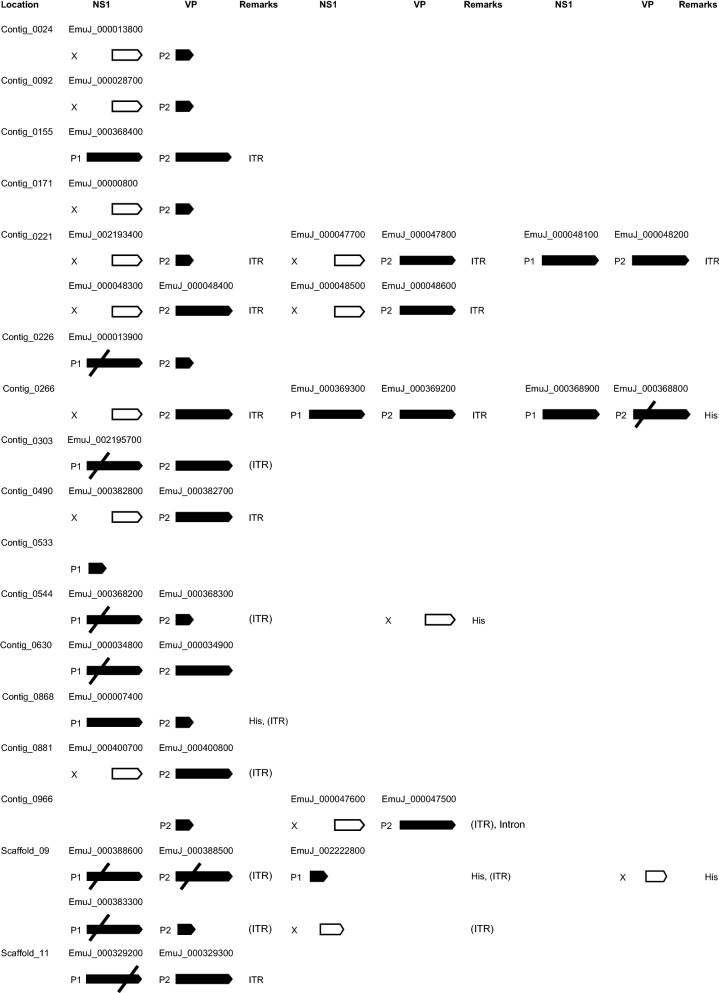
Schematic overview of densovirus genes in *E. multilocularis*. Long black arrows represent full length genes with (crossed out) or without frameshift mutations. Short black arrows represent gene copies with truncated 3′-end, white arrows with truncated 5′-ends. When available, corresponding gene IDs from WormBaseParaSite are shown above the arrows. *Abbreviations*: P1, putative promotor for *Emu*DNV-NS1; P2, putative promotor for *Emu*DNV-VP; X, no promotor; ITR, inverted terminal repeats; (ITR), Remnants of ITRs; His, neighboring histone genes; Intron, containing introns

We next mined the *E. multilocularis* genome for further densovirus integration events and identified a total of 26 loci with high similarity to *Emu*DNV-NS1. All these putative densovirus gene sequences were curated individually and translated into amino acid sequences. BLASTP analyses of the predicted amino acid sequences indicated that all sequences referred to genes encoding full-length or truncated versions of *Emu*DNV-NS1. The longest versions of *Emu*DNV-NS1 (431 amino acids) were encoded by loci on the contigs 0155 (EmuJ_000368400), 0221 (EmuJ_000048100), 0266 (EmuJ_000369300 and EmuJ_000368900) and 0868 (EmuJ_000007400) (Fig. 1). Several gene versions (e.g. EmuJ_000388600) were very similar to those encoding the 431 amino acid protein, but contained frameshift mutations in the N-terminal regions (Fig. 1). In 6 cases, frameshifts had apparently occurred in the gene regions encoding the N-terminal domains of *Emu*DNV-NS1 (Fig. 1). Only 1 gene version (EmuJ_000329200) contained a frameshift in the C-terminal region. All other gene versions were truncated, elven at the 5′-end, two at the 3′-end and one at both ends (see Fig. 1 and Additional file 1: Table S2).

Protein structure analyses of non-truncated versions revealed that in all cases a PPV_E1_C domain and an overlapping Parvo_NS1 domain were present at the C-terminus of the protein, whereas no clear protein domains were predicted within the N-terminal portions. We thus concluded that the predicted *Emu*DNV-NS1 versions were all of parvoviral/densoviral origin. Accordingly, in BLASTP searches against the SWISS-PROT database highest homologies were detectable between *Emu*DNV-NS1 and the non-structural NS1 protein of the *Aedes* densonucleosis virus (23% identical, 42% similar residues) and the *Aedes albopictus* densovirus (24%/43%). In BLASTP searches against the nr database (organism: viruses), high overall homologies (26%/43%) were also found between *Emu*DNV-NS1 and the Non-structural protein 1 of the Infectious hypodermal and hematopoietic necrosis virus (IHHNV), which has been isolated from the blue shrimp, *Penaeus stylirostris* [56].

To detect the ORF(s) for the gene encoding the structural proteins of the capsid (VP), we performed BioEdit six-frame translations of neighboring regions of *Emu*DNV-NS1. We found an ORF 67 nucleotides downstream of many *Emu*DNV-NS1 gene copies encoding a 321 amino acid protein which we designated *Emu*DNV-VP. By BLAST searches we detected 26 versions of *Emu*DNV-VP, 13 of which were full-length (Fig. 1). Two gene versions (EmuJ_000388500 and EmuJ_000368800) were very similar to those encoding the 321 aa protein (93–97% identities and 91–94% identities, respectively) but contained frameshift mutations. In contrast to *Emu*DNV-NS1, where most gene versions were truncated at the 5′-end, 9 of 11 truncated *Emu*DNV-VP versions were truncated at the 3′-end (see Fig. 1 and Additional file 1: Table S2).

When searching neighboring regions of the *Emu*DNV reading frames for inverted terminal repeats (ITRs), we detected ITR sequences of different length, with the longest sequence being located 37 nucleotides downstream of the *Emu*DNV-VP gene version EmuJ_000329300. This ITR sequence was 370 nt long, with a 165 nt stem (89% matches) and a 37 nt loop. BLAST searches revealed that the other identified ITR sequences were shorter, slightly different versions of the same sequence. Additionally, remnants of ITR sequences were detected near several virus genes (see Fig. 1 and Additional file 1: Table S2). The best conserved ITRs were found flanking *Emu*DNV-NS1 EmuJ_000048300 and *Emu*DNV-VP EmuJ_000048400 on contig 0221 with 100% matches with each other and within each ITR (length 228 and 229 nt, stem 95 and 96 nt, loop 37 nt, respectively).

**Fig. 2:**
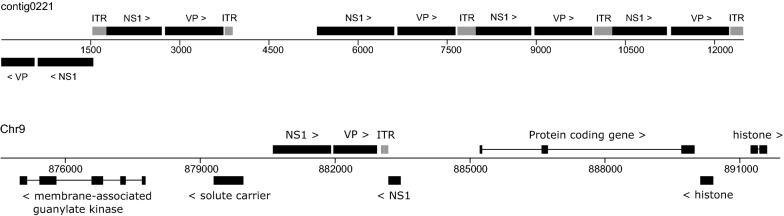
Densovirus loci in the *E. multilocularis* genome. Shown is a schematic representation of the complete contig 0221 and a part of chromosome 9. Numbers indicate position in bp. Black boxes represent exons, lines introns. Arrows indicate gene orientation. *Abbreviations*: NS1, non-capsid protein; VP, capsid protein; ITR, inverted terminal repeats (gray boxes)

Most densovirus loci were located on isolated regions of the genome and several were present as head-to-tail concatemers (Fig. 2). In some cases, densovirus loci were present in protein coding regions of the genome close to histone clusters (Fig. 2). To confirm the correctness of the genome assembly at densovirus sites, we further inspected the gene versions EmuJ_000013900, EmuJ_002195700, EmuJ_000388600 and EmuJ_000329200 by PCR. To this aim, we used primers annealing to the respective *Emu*DNV-NS1 gene version and to a neighboring tapeworm gene, encoding a solute carrier in case of EmuJ_000013900 and EmuJ_000388600 as well as a transcriptional corepressor of histone genes in case of EmuJ_002195700 and EmuJ_000329200 (see Additional file 4: Figure S3). In all cases we succeeded in amplifying PCR products of the expected size and the resulting sequences showed > 99.5% identities to their respective genomic sequences confirming correctness of the genome assembly.

### Expression of densovirus genes in *E. multilocularis*

**Fig. 3:**
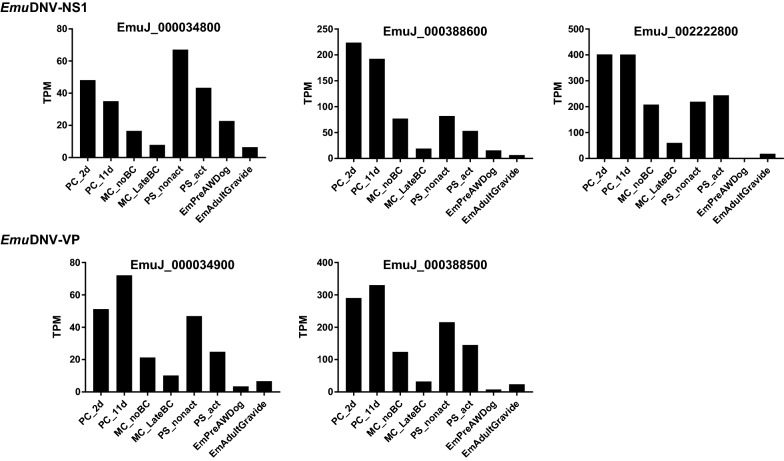
Gene expression of *Emu*DNV-NS1 and *Emu*DNV-VP. Expression is shown in transcripts per million (TPM). *Abbreviations*: PC_2d, primary cells 2 days-old; PC_11d, primary cells 11 days-old; MC_noBC, metacestodes without brood capsules; MC_LateBC, metacestodes with brood capsules; PS_nonact, not-activated protoscoleces; PS_act, activated protoscoleces; EmPreAWDog, pregravid adult; EmAdultGravide, gravid adult

We detected putative TATA boxes and additional potential promoter elements upstream of all *Emu*DNV-NS1 and E*muDNV-VP* genes with a complete 5′-end (Fig. 1). The TATA-box for *Emu*DNV-NS1 was located 53 or 54 nt upstream of the putative start codon and 30 nt upstream of the putative initiation of transcription with the sequence CATTCA (see Additional file 5: Figure S4). The TATA-box for *Emu*DNV-VP was located 34 or 35 nt upstream of the putative start codon and 28 or 29 nt upstream of the putative initiation of transcription with the sequence CACATT. Given that 12 of *Emu*DNV integration loci had identical or highly similar promoter regions, we then investigated whether differential or homogenous expression of these loci occurred. To this end, we mapped existing NGS transcription data [8] to the genome and discarded all reads that mapped to more than one genomic location to only allow unique assignments for re-analysis of the expression profiles. Interestingly, while 14*Emu*DNV-NS1 and 24 *Emu*DNV-VP versions had putative promotor elements, only 3 *Emu*DNV-NS1 and 2 *Emu*DNV-VP gene versions were actually expressed (cut off: 10 TPMs) (Fig. 3). All expressed versions of *Emu*DNV-NS1 were either truncated at the 3′-end (EmuJ_002222800) or contained N-terminal frameshift mutations (EmuJ_000034800 and EmuJ_000388600). Of the expressed *Emu*DNV-VP versions, one was a full-length version (EmuJ_000034900) and one had an N-terminal frameshift mutation (EmuJ_000388500). These data indicated that the majority of *Emu*DNV loci were transcriptionally silenced.

To verify the transcriptomic data by RT-PCR, *Emu*DNV-NS1 was amplified from cDNA of 2-day-old *E. multilocularis* primary cell preparations using primers binding to four *Emu*DNV-NS1 gene versions without mismatches (EmuJ_000034800, EmuJ_000388600, EmuJ_002195700, EmuJ_000329200) and to further 8 gene versions with mismatches (0–5 mismatches per primer). As expected, no PCR products were obtained from RT-negative cDNA preparations. For RT-positive cDNA preparations, on the other hand, a clear band of the expected size (*c.*1100 bp) was obtained and cloned. Eight of the obtained clones were analyzed and six of them yielded identical sequences. The other 2 sequences differed in only 1 nucleotide from the 6 sequences and were considered variations of the same sequence. The 1103 bp long partial sequence (deposited at the EMBL Nucleotide Sequence Database under the accession number LR029140) showed 99.8% homologies (2 mismatches) to the *Emu*DNV-NS1 version EmuJ_000388600 whereas at least 16 mismatches were observed to all other DNV-NS1 loci on the genome. We therefore concluded that the obtained sequence originated from the *Emu*DNV-NS1 version EmuJ_000388600, confirming gene expression of *Emu*DNV-NS1 in *E. multilocularis* and indicating that the gene versions EmuJ_000034800, EmuJ_002195700, EmuJ_000329200 are not or very lowly expressed.

### Densovirus gene expression in *E. multilocularis* germinative cells

**Fig. 4:**
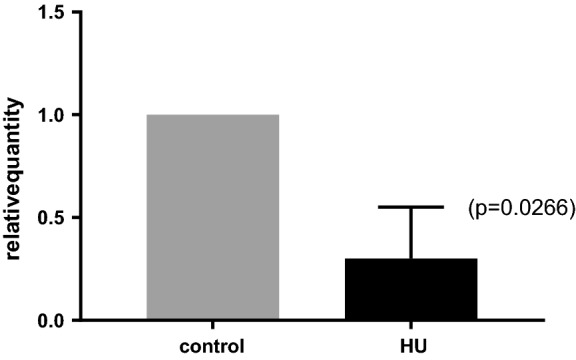
Gene expression of *Emu*DNV-NS1 after depletion of germinative cells. qRT-PCR was performed for *Emu*DNV-NS1 (EmuJ_000034800, EmuJ_000388600 and EmuJ_000329200) with cDNA from metacestodes treated with hydroxyurea (HU) and untreated controls. The experiment was performed with 3 technical and 3 biological replicates. Expression was normalized to control. Error bar of HU-sample is 1 SE

According to RNA-Seq data, all *Emu*DNV genes showed a transcription profile typical of germinative cell-specifically expressed genes with high expression in *E. multilocularis* primary cell preparations (metacestode cell preparations with 80% germinative cell content [51]) and lower expression in the metacestode and protoscolex stages (Fig. 3). To further investigate the *Emu*DNV gene expression profiles, we made use of a method for specific deprivation of *E. multilocularis* germinative cells in metacestode vesicles which we had previously introduced [51]. To this end, we specifically eliminated the germinative cell population from *in vitro* cultivated metacestode vesicles, which otherwise remained intact. We then performed qRT-PCR against *Emu*DNV-NS1 (EmuJ_000034800, EmuJ_000388600, EmuJ_000329200) on vesicles without or with germinative cells. As shown in Fig. 4, the expression of *Emu*DNV-NS1 was significantly reduced in vesicles after treatment with HU, indicating that densovirus genes are specifically or at least preferentially expressed in the parasite’s germinative cell population.

### Identification of densovirus integration sites in other tapeworm species

**Fig. 5:**
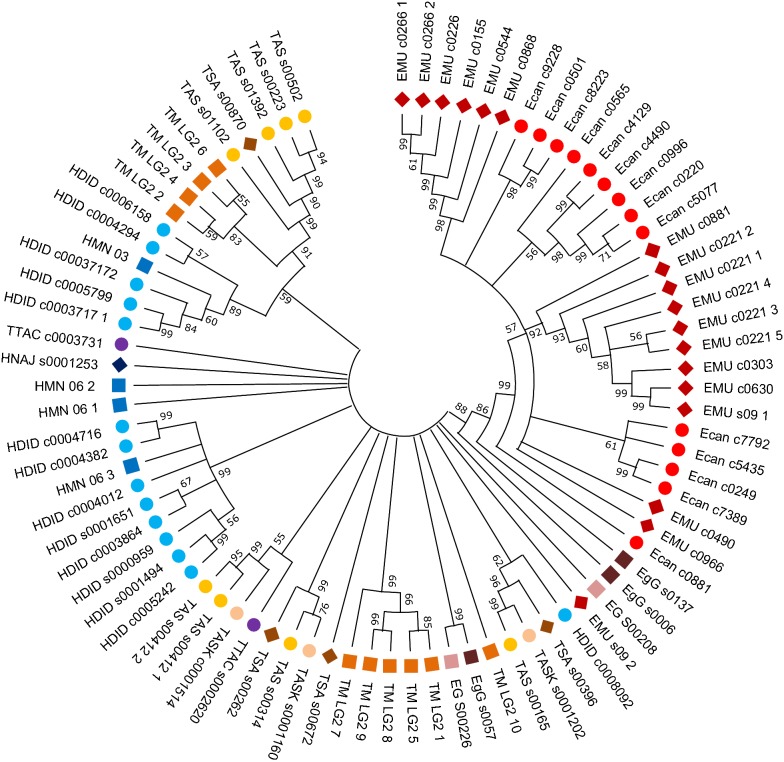
Phylogenetic analysis of densovirus NS1 genes. The neighbor-joining tree was constructed with nucleotide sequences (coverage > 50%) using MEGA-X. Numbers at the branching nodes indicate their percentage of appearance in 1000 bootstrap replications. Branches reproduced in less than 50% of replications were collapsed. *Abbreviations*: Ecan, *E. canadensis*; EgG, *E. granulosus* (PRJEB121); EG, *E. granulosus* (PRJNA182977); EMU, *E. multilocularis*; TTAC, *H. taeniaeformis*; HDID, *H. diminuta*; HMN, *H. microstoma*; HNAJ, *H. nana*; TASK, *T. asiatica* (PRJEB532); TAS, *T. asiatica* (PRJNA2998719); TM, *T. multiceps*; TSA, *T. saginata*

In search for densovirus NS1 gene sequences in other cestodes we surveyed the genomes of *D. latus*, *E. canadensis*, *E. granulosus*, *H. taeniaeformis*, *H. diminuta*, *H. microstoma*, *H. nana*, *M. corti*, *S. solidus*, *S. erinaceieuropaei*, *T. asiatica*, *T. multiceps*, *T. saginata* and *T. solium* alongside with *E. multilocularis*, and included *S. mansoni* as a trematode example (for details on genomes see Additional file 1: Table S1). With BLAST searches we detected a total of 211 putative NS1 gene sequences, mostly in the genomes of *H. diminuta* (*n* = 37), *E. canadensis* (*n* = 24), *E. multilocularis* (*n* = 23) and *T. asiatica* (PRJNA299871) (*n* = 23). Further sequences were detected in the genomes of *T. multiceps* (*n* = 21), *H. microstoma* (*n* = 19), *H. nana* (*n* = 17), *T. asiatica* (PRJEB532) (*n* = 12), *T. saginata* (*n* = 12), *E. granulosus* (*n* = 6 each in PRJEB121 and PRJNA182977), *H. taeniaeformis* (*n* = 4), *T. solium* (4) and *S. mansoni* (*n* = 3) (see Additional file 1: Table S3). No putative NS1 gene sequences which fulfilled the inclusion criteria were identified in the genomes of *D. latus*, *M. corti*, *S. solidus* and *S. erinaceieuropaei.* Phylogenetic analysis of sequences with > 50% coverage of *Emu*DNV-NS1 EmuJ_000388600 showed clustering of sequences within and between species (Fig. 5), indicating expansion of densovirus sequences also after platyhelminth diversification.

## Discussion

One of the most striking features of the genomes of parasitic flatworms (i.e. trematodes and cestodes) when compared to free-living flatworm species and all other animals is the absence of true orthologues of the common stem cell markers *piwi* and *vasa* [8, 11, 57], which are important components of the germline multipotency program [13] and are usually expressed by germline stem cells to protect their genomes against MGE and viruses [12].Circumstantial evidence for the absence of a canonical piwi/piRNA pathway in parasitic flatworms was also obtained by several sequencing projects concerning trematode and cestode small RNAs, which identified several microRNAs or endo-siRNAs but did not yield any indications for the presence of piRNAs in these organisms [58, 59]. This led to important questions on alternative mechanisms that are employed by parasitic flatworms to protect their genomes against transposons [11]. Cestode genomes contain a number of repeats with characteristics of transposable elements such as GYPSY class of LTR retrotransposons or *Merlin* DNA transposons [8, 16]. Furthermore, we recently identified a terminal repeat retrotransposon in miniature (TRIM) family which is massively expressed in germinative cells of taeniid cestodes [17]. Hence, it is expected that cestodes employ MGE protective mechanisms other than the piwi/piRNA pathway [11] but the molecular nature of these mechanisms is elusive so far. Of particular interest in this regard would be the identification of transposons in parasitic flatworm genomes which show features of silencing.

In the present work, we provide evidence for the presence of densovirus genes in the genomes of cestodes. The elements we identified displayed clear structural homologies to parvo- and densovirus elements found in other organisms such as reading frames encoding proteins with similarity to non-structural (NS1) and virus capsid proteins which are flanked by ITR. The presence of densovirus sequences in the vicinity of histone clusters, together with confirmation of the genome assembly at selected integration sites by PCR analysis, clearly indicate true integration events during cestode genome evolution. The presence of densovirus-related sequences in 13 of 17 analyzed cestode genomes indicates widespread endogenization of densoviruses in cestodes. Strongly varying numbers of densoviral sequences detected in the analyzed species might not correspond to different numbers of integration events, but could be caused by the different qualities of the genome assemblies. Many identified densoviral sequences are located on small contigs or near repetitive sequences, such as histone clusters. As repetitive sequences are generally difficult to assemble and often collapsed in the genome assembly, it is likely that the number of detected densoviral sequences is influenced by the quality of the genome assembly and the real number of sequences in the genome and might be higher. Additionally, densovirus sequences could appear to be truncated because the contig does not continue at this position which would lead to an underestimation of the number of complete densoviral sequences.

Although all densoviral genes with a complete 5′-end have intact promotor elements, the majority of them appear to be transcriptionally silent. According to transcriptome data only three densovirus loci are transcriptionally active. RT-PCR confirms expression of the *Emu*DNV-NS1 version EmuJ_000388600. In contrast, we did not obtain sequences for three other *Emu*DNV-NS1 versions with equal primer binding properties suggesting that they are not or relatively lowly expressed. This is in accordance with the transcriptome data that show no expression for two of them and comparatively low expression levels for the third. The presence of intact promotor elements together with apparent silencing of most densovirus loci indicates a specific silencing mechanism. We propose that epigenetic silencing might be the underlying mechanism. DNA methylation was recently detected in cestodes [60] and has already been suggested as a mechanism for silencing of parvovirus B19 [61]. Further studies, for example comparison of methylation patterns of actively transcribed and silent densovirus loci, are required to evaluate a potential role of DNA methylation in silencing of densoviruses and possibly other mobile genetic elements in cestodes.

Phylogenetic analysis of NS1 sequences in cestodes indicates a spread of densoviral sequences within species. Although the current cestode genome assemblies did not allow us to specifically determine whether a given densovirus locus has integrated into the *E. multilocularis* genome after the separation of taeniid cestode species or earlier, our phylogenetic analyses nevertheless indicate that densoviruses were still actively spreading after the separation of *E. multilocularis* and *E. granulosus*. To address the question if densoviruses in cestodes are still able to replicate and spread, we examined if densoviral genes are expressed in germinative cells of *E. multilocularis.* Transcriptome data and qRT-PCR strongly indicate specific or preferential expression in germinative cells which provides an explanation for maintenance of densoviral sequences in the parasiteʼs germline-like cell population. It is thus likely that the other cestodes also express densoviral genes in their germinative cells. Parvoviral NS1 activities, such as endonuclease and helicase activity, are required for parvoviral DNA replication [62, 63]. However, none of the expressed *Emu*DNV-NS1 gene versions contain a complete and intact N-terminal domain without truncation or frameshift mutation suggesting that no active NS1 protein is available for densovirus replication in *E. multilocularis*. It is therefore questionable whether contemporary horizontal transmission events of endogenous densoviruses are possible in cestodes.

Interestingly, densovirus-based vectors have already successfully been used for genetic manipulation of insect cells and mosquitoes [24, 26]. The advantage of these manipulation systems is that, in contrast to adenoviruses or lentiviruses, no complete virus particles have to be used for obtaining integration competent vector constructs. Instead, densovirus systems can be introduced simply by plasmids into target cells and utilize the activity of NS1 genes for genomic integration [24, 26]. On the basis of the densovirus sequences we identified in this study it should be possible to reconstruct functionally active NS1 loci and to utilize terminal repeat information for successful genetic manipulation of *E. multilocularis* in the near future. Experiments towards this aim are currently underway.

## Conclusions

Although tapeworms lack a canonical piRNA-pathway, their germline has to be protected against the activities of transposons in their genomes. Investigating possible transposon silencing mechanisms first requires comprehensive information on mobile genetic elements in these organisms. The data presented herein show integration of densovirus-related elements in a large number of tapeworm species. Transcriptome data and RT-PCR further indicates active transcription of some densovirus gene versions in *E. multilocularis*, whereas most remain transcriptionally silent. Further study of active and silent elements will provide first clues for transposon silencing mechanisms in *E. multilocularis* and other cestodes. Our results further point to the possibility of utilizing densovirus-based vectors for genetic manipulation of *E. multilocularis* and other cestodes.

## Supplementary information

**Additional file 1: Table S1.** Overview of analyzed tapeworm genomes. **Table S2.** Densovirus sequences in *E. multilocularis*. **Table S3.** Densovirus NS1 gene sequences in tapeworm genomes.

**Additional file 2: Figure S1.** Schematic overview of the bioinformatics workflow.

**Additional file 3: Figure S2.** Alignment of densovirus NS1 sequences.

**Additional file 4: Figure S3.** Densovirus integration sites in the *E. multilocularis* genome.

**Additional file 5: Figure S4.** Promotor regions of *Emu*DNV-NS1.

## Data Availability

Data supporting the conclusions of this article are included within the article and its additional files. The sequence of *Emu*DNV-NS1 is available in the EMBL Nucleotide Sequence Database under the accession number LR029140 (https://www.ebi.ac.uk/). The genome datasets analyzed during the present study are available at WormBaseParaSite (https://parasite.wormbase.org), the RNA-Seq reads in the EMBL Nucleotide Sequence Database (https://www.ebi.ac.uk/, accession numbers ERS094035, ERS094036, ERS094037, ERS094038, ERS094039, ERS016464, ERS018054 and ERS018053).

## Abbreviations

BLAST: basic local alignment search tool
DNV: densovirus
EdU: 5-ethynyl-2′-deoxyuridine
HU: hydroxyurea
ITR: inverted terminal repeat
MGE: mobile genetic element
PBS: phosphate-buffered saline
RT: reverse transcriptase
TPM: transcripts per kilobase of exon per million transcripts mapped
