# Supplementary figures and images for: Evidence for densovirus integrations into tapeworm genomes
Source: Parasit Vectors. 2019 Nov 27;12:560. doi: 10.1186/s13071-019-3820-1 (PMC6880638; doi:10.1186/s13071-019-3820-1)

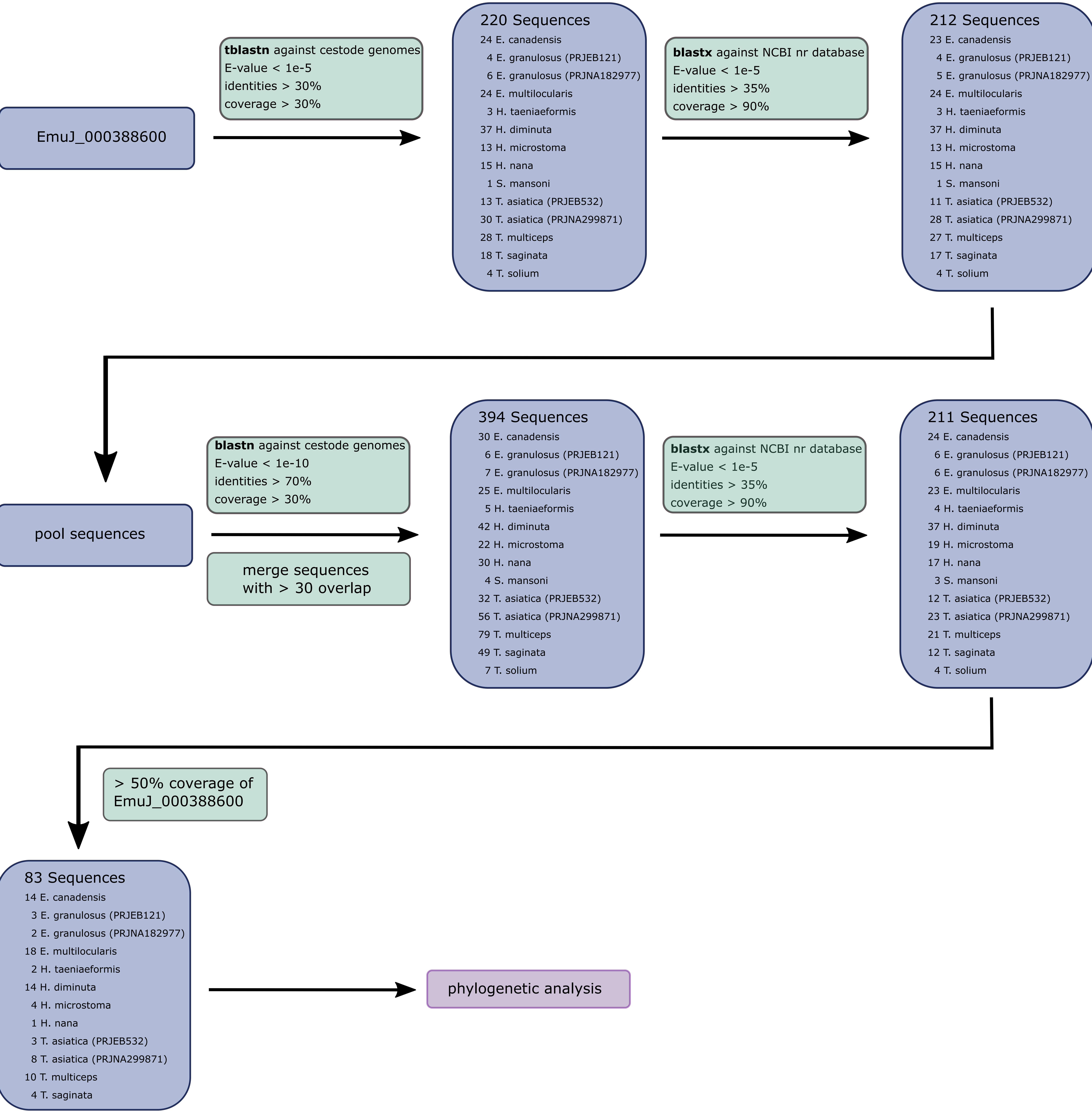

Additional file 2: Figure S1. Schematic overview of the bioinformatic workflow

Supplement: Supplementary file 2 — Additional file 2: Figure S1. Schematic overview of the bioinformatics workflow. [file 13071_2019_3820_MOESM2_ESM.pdf]
