# Supplementary material for: Evidence for densovirus integrations into tapeworm genomes
Source: Parasit Vectors. 2019 Nov 27;12:560. doi: 10.1186/s13071-019-3820-1 (PMC6880638; doi:10.1186/s13071-019-3820-1)
Supplement: Supplementary file 3 — Additional file 3: Figure S2. Alignment of densovirus NS1 sequences. [file 13071_2019_3820_MOESM3_ESM.pdf]

```
EmuJ_000388600.1  ------------------------------------------MHVIFQSSPNNSVRKMERIIEE------------------------------------ 22
P27454.1          MEYGLISKFYMDHWRWKIREKHKVENNLLSTYKYILNFHYHMVHPYIRSTKGGPNRVMNSVCVEHSPCEHGNLFCECIYCWEHDGQCRGRK--LDLGAST 98
Q90185.1          ------------------------------------------STEYIRGINEGRVSSMESVCSEHSPCEHGNIECECIFCWEHDAQCKSRRRELDLGEPT 58

EmuJ_000388600.1  -------------------CGVPLQAVPDI-------------KMTKQLVRNVTALIRYMRGRGEVVA--------------------------TDDHFD 64
P27454.1          GIERRLANDNQQPGLSDLYCTETIHLATAIPERRTIDRENYVKDFAGQTVGDLYPQLQGSTGASEPIDFAFPTVGSGSWEILVRESHKHFEPNYTEEAYQ 198
Q90185.1          GSERGMANNYEQSRNEDLYCTETVPSSAAL-QANTITERDIREDFTDQTVDNIYPQLHSGSRASEQLEFAFPTIGTRSWEILIRQSYEHLKPDYKEEDFQ 157

EmuJ_000388600.1  HFLQ-----------------------------------------------------VATVSLEWPNCSVIPSENR--------RMNKSAKEEDKREVKR 103
P27454.1          SHIRSVRRRLFPEETMDNNGSQASTTEMLRDAVQRCGFEGPPNSPSENNRDGIDGTCISTVDIQ-SNCIVNAHCPKQGTSNQTNKRKKSTDTTESSGSKK 297
Q90185.1          SHIRRVRRQLFPEKTMDNNGSQASTTQMLRDDIERCGIESIADSASEDNGDVDGTCISTVDIQ-GNCIVNAHGNEQATGSKT-RKIRATTTPESSESKK 255

EmuJ_000388600.1  QKY---------------IDLAEEIMRRKVRSMNDMNKKFTY----------QETFRLMA---------------------------------------- 138
P27454.1          NKSSNYQQNLQEQGSTS-ISDTIDIVDGELDGSTGSNRETAYYTFVLHKNNVKEDWRYIATTRAKQAPSFITFDHGDHIHILFSSSNTGGNSTRVRTRIT 396
Q90185.1          HKGSNNQQNIQEQSSTTAIADGHDIVDGELDGRSESNRETAYYTFVLHKNNCKEDWRYIATTRAKQAPSFITFDHGDHIHILFSSSNTGGNSTRVRTRIT 355

EmuJ_000388600.1  --------------------DYEQSYNM-IVRKALETVRTM-NVAHQRATDYMDLLKEELDN-------VRNGCPSHL---CAYPKNHSGPSRKES---- 202
P27454.1          KFLSATSAGSAEATITFSKVKFLRNYILYCIRYGIETVNIYGNKIQQQLTEAMDTFKILFENRDPNDVILEAGCKLYHEEKKDNKQKRCGQRKQQNLTDI 496
Q90185.1          KFLSATSAGNAEATITFSKVKFLRNYILYCIRYGIETVNIYGNKIQQQLTEAMDTFKVLFENRDPNDVILDAGCKLYHEEKKEYQQKRCGQRKQQNLTDI 455

EmuJ_000388600.1  ----------------------------------------------------------------------------------------IQWLEDMFSANE 214
P27454.1          ILEKIKEKKITTAQQWENQIEPEFKIQLMKEFGLNVDSYVTRIVRIERTRIQQLIKAKTLTEIMLEILNDEYIKHFTPGEDNSKTAKCIEWIEYLFKENN 596
Q90185.1          ILDKIKEKKITTAQQWENIIEPEFKIQLMKEFGLNVDSYVQRIVRIERTRIQQIIKSKTLTEIMVEILNEEYIKNFTPGEDNSKLQNIIQWIEYMFKENN 555

EmuJ_000388600.1  IAVVDFAITLRIIMNCEDEKINTLVLYGPTNTGKSLICRLTTTFLEHGSVMRRQEASAFAYENLLNRKVALMEEPRICAANQQDLKQILGGEPFEVHIKY 314
P27454.1          INIIHFLAWNEIIKTKRYKKINGMVLEGITNAGKSLIILDNLLAMVKPEEIPRERDNSGFHLDQVPGAGSILFEEPMITPVNVGTWKLLLEGKTIKTDVKN 696
Q90185.1          INIIHFLAWNEIIKPKRYKKINGMVLEGITNAGKSLIILDNLLAMVKPEEIPRERDNSGFHLDQLPGAGSVLFEEPMITPVNVGTWKLLLEGKTIKTDVKN 655

EmuJ_000388600.1  QNPDLLERLPVIVTTNEPLGVRLSDVDAAAIEGRCKIYTLDKQI--------CNANIDGSVPA-PPYKLCACDMAHLLLP----IYELLAL--------- 392
P27454.1          KDKEPIERTPTWITTATPITNNIDMNETSQILQRIKLYILKKSIQHRDDKYTINAQIQNKLISRPPTLIEPIHMAIVFIKNFTKIYNLIAEEDKAHTVNE 796
Q90185.1          KDKEPIERTPTWITTATPITNNVHMNETSQILQRIKLYIFKKSIQHREDKYTINAQIQNKLISRPPGLVEPIHMAIVFVKNFKEIYKLIEEEDNAHTVNE 755

EmuJ_000388600.1  ----------------------------------------------------- 392
P27454.1          KAIQINNEVKEEAESWQTALQWTMTENNEEQNENETQALEDQVLELAKEQATT 849
Q90185.1          KAIRLSEEAKQEAEEWQTALQWNTMEEEQKENEKQT---EDQDKESEKETATQ 805
```

Additional file 3: Figure S2. Alignment of densovirus NS1 sequences. Identical amino acids are shown white on black background, biochemically similar amino acids are shown on grey background. The black line indicates the position of the Parvo_NS1 domain (PF01057.17) in EmuJ_000388600. EmuJ_000388600: *E. multilocularis* densovirus NS1; P27454.1: *Aedes densonucleosis virus* NS1; Q90185.1: *Aedes albopictus parvovirus* NS1.
