# Supplementary material for: Evidence for densovirus integrations into tapeworm genomes
Source: Parasit Vectors. 2019 Nov 27;12:560. doi: 10.1186/s13071-019-3820-1 (PMC6880638; doi:10.1186/s13071-019-3820-1)
Supplement: Supplementary file 4 — Additional file 4: Figure S3. Densovirus integration sites in the E. multilocularis genome. [file 13071_2019_3820_MOESM4_ESM.pdf]

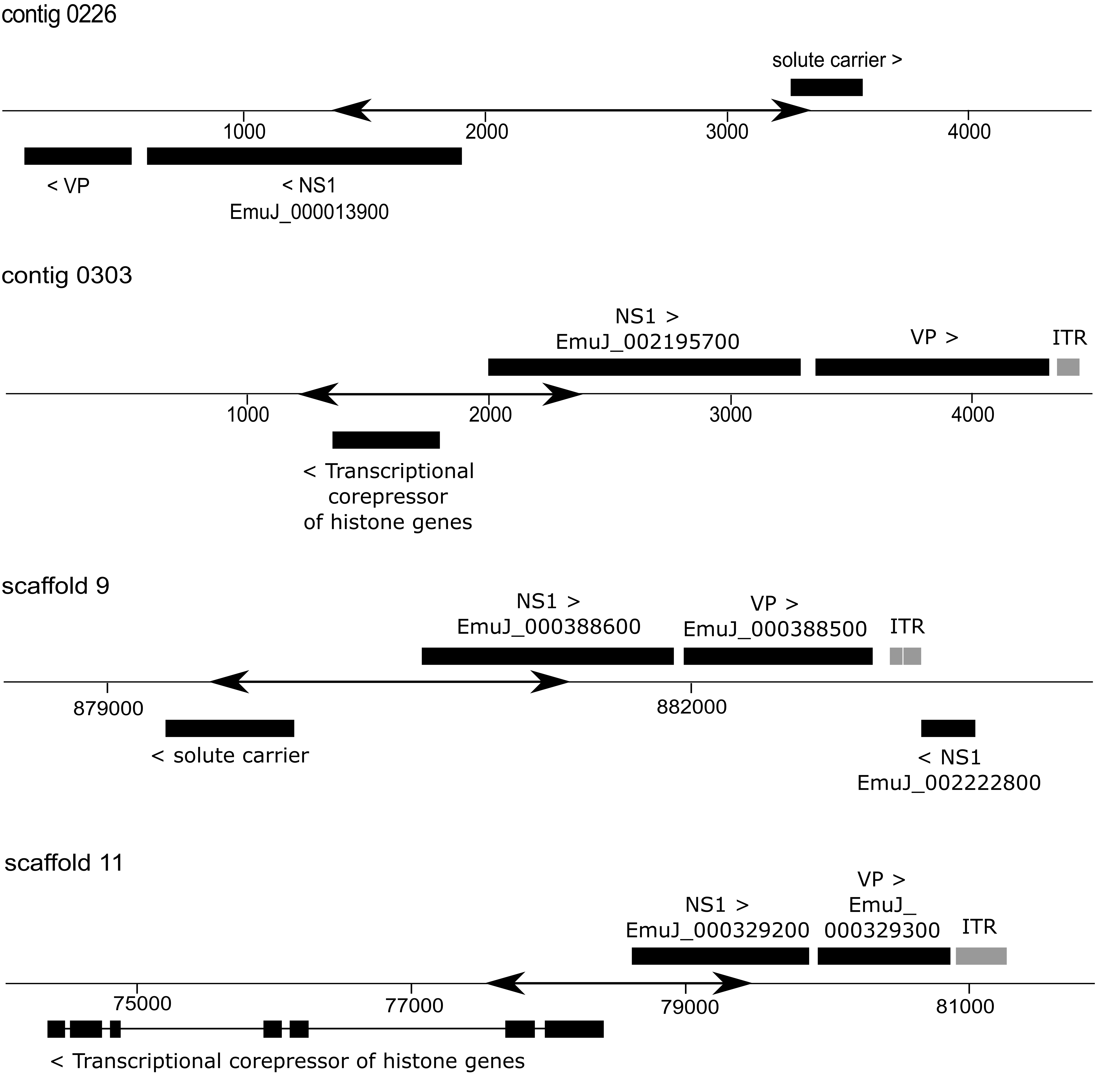

Additional file 4: Figure S3. Densovirus integration sites in the *E. multilocularis* genome. Shown is a schematic representation of the complete contigs 0226 and 0303 and parts of scaffold 9 and 11. Exons and ITRs are represented by black and grey boxes, introns by lines. Arrowheads indicate gene orientation. Double headed arrows show sequenced genome regions. NS1: non-capsid protein 1; VP: capsid protein; ITR: inverted terminal repeat.
