# Supplementary material for: Evidence for densovirus integrations into tapeworm genomes
Source: Parasit Vectors. 2019 Nov 27;12:560. doi: 10.1186/s13071-019-3820-1 (PMC6880638; doi:10.1186/s13071-019-3820-1)
Supplement: Supplementary file 5 — Additional file 5: Figure S4. Promotor regions of EmuDNV-NS1. [file 13071_2019_3820_MOESM5_ESM.pdf]

```
GCAGCTGACTAACGATGCTTTGTTTTAATAGAGTATAAATATGGGCGATTTTCGTGGAGCTGACATTCAGCTTCCGGACG   EmuJ_000388600
GTAGCTGACTAACGATGCTTTGTTTTAATGGAGTATAAATATGGGCGGTTTTCGTGGAGCTGACATTCAGCTTCCGGACG   EmuJ_000369300
: ::::::::::::::::::::::::::: ::::::::::::::::: ::::::::::::::::::::::::::::::::

TGTTGTGATGTCGCAGACCTCTCTTGCCGGTTTCACAAGGAGTTACCATACATTTATCTTGCGGTTTTATACCAGAACAC   EmuJ_000388600
TGCGAA-ATGTCGCAGACCACTCTGAGCGGTTTTACCAGAACCTACTACACCTTCATTTTAAGGCAGTATACCGGAAGAC   EmuJ_000369300
::     :::::::::::: ::::   :::::: :: :: :  ::: : :: :: :: ::  ::   :::::: ::: ::
        M  S  Q  T  T  L  S  G  F  T  R  T  Y  Y  T  F  I  L  R  Q  Y  T  G  R

CCAATGCTCTATCGGAAGTTCTGTATAC----------GATCACATGCACGTTATCTTCCAAAGCAGCCCCAATAACTCG   EmuJ_000388600
CCGATGCTATGTCTGAGGTGCTTTATACGGAACACGATGATCACATGCACGTGATCTTCCAAAGCAGTACCACGAACTCG   EmuJ_000369300
:: ::::: : :: :: :: :: :::::          :::::::::::::::::::::::::::::  :::  ::::::
P  D  A  M  S  E  V  L  Y  T  E  H  D  D  H  M  H  V  I  F  Q  S  T  T  N  N  S
```

Additional file 5: Figure S4. Promotor regions of *Emu*DNV-NS1. Alignment of the promotor regions of EmuJ_000388600 and EmuJ_000369300. Identical nucleotides are indicated by points. Putative promotor elements (TATA-Box, initiation of transcription and DPE-like box) are underlined. The amino acid sequence of EmuJ_000369300 is shown below the alignment. The sequence arising from an alternative start codon is underlined.
